# Pharmacological Basis for Use of* Selaginella moellendorffii* in Gouty Arthritis: Antihyperuricemic, Anti-Inflammatory, and Xanthine Oxidase Inhibition

**DOI:** 10.1155/2017/2103254

**Published:** 2017-01-05

**Authors:** Ping Zhao, Ke-li Chen, Guo-li Zhang, Guang-rui Deng, Juan Li

**Affiliations:** ^1^Key Laboratory of Ministry of Education on Traditional Chinese Medicine Resource and Compound Prescription and College of Pharmacy, Hubei University of Chinese Medicine, Wuhan, Hubei 430065, China; ^2^Department of Pharmacy, Huanggang Hospital of Traditional Chinese Medicine, Huanggang 438000, China; ^3^State Key Laboratory of Dao-Di Herbs, National Resource Center for Chinese Materia Medica, China Academy of Chinese Medical Sciences, Beijing, China

## Abstract

This study was aimed at evaluating the effects of* Selaginella moellendorffii* Hieron. (SM) on gouty arthritis and getting an insight of the possible mechanisms. HPLC method was developed for chemical analysis. The paw oedema, the neutrophil accumulation, inflammatory mediators, lipid peroxidation, and histopathological changes of the joints were analyzed in gouty arthritis rat model, and the kidney injury and serum urate were detected in hyperuricemic mice. Pharmacokinetic result demonstrated that the main apigenin glycosides might be quantitatively transformed into apigenin in the mammalian body. Among these compounds, the apigenin exhibited the strongest effect on xanthine oxidase (XOD). SM aqueous extract has proved to be active in reducing hyperuricemia in dose-dependent manner, and the levels of blood urea nitrogen (BUN) and creatinine (Cr) in high dose group were decreased significantly as compared with hyperuricemic control group (*P* < 0.01). The high dose of SM extract could significantly prevent the paw swelling, reduce gouty joint inflammatory features, reduce the release of IL-1*β* and TNF-*α*, lower malondialdehyde (MDA) and myeloperoxidase (MPO) levels, and increase superoxide dismutase (SOD) level (*P* < 0.01). For the first time, this study provides a rational basis for the traditional use of SM aqueous extract against gout in folk medicine.

## 1. Introduction

Gout, a MSU crystals-induced acute inflammatory arthritis, is a metabolic disorder with a worldwide distribution and characterized by hyperuricemia and MSU deposition in the articular cavity [1]. The gout occurrence is induced by the interaction of genetic and environmental factors. The latter consists of obesity, purine rich diet, high blood pressure, and alcohol consumption [2, 3]. During hyperuricemia, oversaturation of serum urate and formation of MSU crystals deposited in the joints activate an inflammatory cascade, followed by acute inflammatory response such as edema, swelling, nociceptive behaviors, and neutrophil infiltration [4–7]. Furthermore, MSU crystals induce the release of various inflammatory mediators such as cytokines (IL-1*β*, TNF-*α*), matrix metalloproteinases, chemokines, prostaglandin E2 (PGE-2), and oxygen radicals [8–10], leading to the tissue and bone damage.

Now, the drug treatments of gout are allopurinol [11], inhibiting XOD activity, probenecid promoting the renal excretion of uric acid (UA), colchicine inhibiting neutrophil infiltration [12], glucocorticoids, and nonsteroidal anti-inflammatory drugs mainly inhibiting cyclooxygenase (COX) enzyme activity [13, 14]. Although these drugs are generally effective, they also present adverse effects or may induce symptom rebound [15]. Thus, the search for new effective therapy with anti-inflammatory and urate-lowering activities, including XOD inhibitors, has motivated several recent studies [16].

*Selaginella moellendorffii *Hieron., which belongs to the family Selaginellaceae, is a traditional Chinese folk herb with extremely abundant resources. It has been used as ethnic drug for treatment of bleeding and chronic inflammation, such as arthritis, gonorrhea, hepatitis, and mastitis [17, 18]. Previous phytochemical studies about SM focused on constituents including bioflavonoids [19], lignans [20, 21], and phenols [22]. Flavonoids and phenols make the medicinal plants ideal candidate for hyperuricemia and gouty arthritis, with their activity of antioxidant, anti-inflammatory, and XOD inhibitory. Also, it is reported that ginkgetin separated from SM could inhibit adjuvant-induced arthritis in rat with no obvious adverse effect [23], SM extracts had inhibition activity in XOD, lipoxygenase (LOX), and COX2 [24, 25]. However, although substantial evidence now exists to support beneficial pharmacological action for SM, the antigout activities and pharmacological mechanism are still unknown due to the coexistence of multiple bioactive components and limited approaches for preparing extracts from SM.

In the present study, we investigated the effect of aqueous SM extract on hyperuricemia and gouty arthritis and get an insight of the possible mechanism and the main metabolites.

## 2. Materials and Methods

### 2.1. Animals

Male Sprague-Dawley rats (180–220 g) and male Swiss mice (18–22 g) were bought from the Hubei Provincial Center for Disease Control and Prevention, Wuhan, China. All animals were kept in a specific pathogen-free animal room under controlled conditions at the temperature 24 ± 2°C and humidity 55 ± 15% with a 12 h light-dark cycle. Animals were allowed to acclimatize to the environment for 1 week before experiment. The treatment and care for the animals were accorded with the internationally approved protocols for laboratory animals.

### 2.2. Materials

Intact plants of SM were purchased from Yi Chang in Hubei province, China. The samples were identified by authors (*Prof.* Ke-li Chen). Specimens of these plants were deposited in the herbarium, Hubei University of Chinese Medicine, China.

Sodium urate, potassium oxonate, adenine, allopurinol, colchicine, XOD, and xanthine were purchased from Sigma-Aldrich, USA. The UA, BUN, Cr, MPO, MDA, SOD kits, TNF-*α*, and IL-1*β* ELISA kits were purchased from Nanjing Jiancheng Bioengineering Institute, Nanjing City, China.

### 2.3. Extracts Preparation

Plant material was pulverized and screened through mesh size of 24. Then, 2 kg of the collected powder was immersed in 20 L distilled water. The solution was heated to 100°C for 1 h, for three times. The combined aqueous extract was filtered and condensed into a final concentration of 1 g raw material/mL, which was used for the subsequent experiment.

### 2.4. HPLC Analysis of SM Extract

The detailed method of HPLC analysis could be seen in literature [25]. Briefly, HPLC analysis of extracts was performed on a Dionex HPLC system with P680 Pump, a Agilent ZORBAX SB-C18 (4.6 mm × 250 mm, 5 *μ*m), and a UVD 170 U variable wavelength UV-Vis detector. Data were collected and processed using “Chromeleon version 6.0” software. The mobile phase consisted of methanol (A) and water (B). The gradient program was as follows: 5–25% A in 0–30 min, 25–50% A in 30–80 min, 50–75% A in 80–97 min, and 75–100% A in 97–100 min. The flow rate was 1.0 mL/min and column temperature was maintained at 30°C. The injection volume was 10 *μ*L. The detector was set at 330 nm for acquiring chromatograms.

### 2.5. Determination of SM Metabolites in the Plasma

SM extracts (16 g/kg body weight of raw medicinal herbs) were orally administered to rats by direct stomach intubation. The rats were killed 0, 30, and 60 min after the administration by withdrawing blood by heart puncture using heparinized needles and syringes under anesthesia with diethyl ether. The plasma was immediately obtained from the collected blood by centrifugation at 1600 ×g for 15 min at 4°C. Then the plasma was involved in a single protein precipitation step with methanol. The supernatant was condensed to 100 *μ*L for HPLC analysis. The mobile phase was methanol: 0.2% phosphate buffer (55 : 45). The flow rate was 1.0 mL/min and column temperature was kept at 30°C. Samples were detected by UV-detector at 350 nm for acquiring chromatogram and the injection volume was 20 *μ*L.

### 2.6. Inhibition of XOD

As described by Cos et al. [26], XOD activity was determined spectrophotometrically at 290 nm. The assay mixture, containing 40 mM phosphate buffer, pH 7.5, 0.2 mM ethylenediaminetetraacetic acid (EDTA), and 5–50 *μ*M xanthine, was incubated in a quartz cuvette at 37°C for 2 min. The reaction was started by the addition of 10 mU XOD and the increase value in absorption at 290 nm was recorded at 5 s intervals for 5 min. The compounds were dissolved in DMSO to obtain various concentration of solutions. The procedure was repeated with the addition of compounds at a range of concentrations. In any appropriate long period of time, absorbance was increased linearly with time, and the slope was reaction rate (dA/min). The greater slope shows the stronger enzyme activity. First, inhibition rate against XOD of all kinds of compounds was determined in the concentration of 100 *μ*M, and then IC_50_ values were further measured on the samples whose inhibition rate was more than 50%.

### 2.7. Animal Model of Hyperuricemia in Mice

In order to evaluate the antihyperuricemic activity of SM, an experimental model of hyperuricemia induced by potassium oxonate was adopted, with modifications [27]. Mice were divided into 6 experimental groups (*n* = 10). In group 1, the normal group, each animal received only normal saline as vehicle. In group 2, the hyperuricemic control group, potassium oxonate (250 mg/kg) was administrated intraperitoneally and adenine (300 mg/kg) was administrated intragastrically. In groups 3, 4, 5, and 6, each animal received the same dose of potassium oxonate and adenine 1 h before administration of SM extracts (30 g/kg, 60 g/kg, and 90 g/kg raw medicinal herbs, resp.) and 20 mg/kg allopurinol, respectively. The samples were administrated to corresponding groups by oral gavage once a day for 2 weeks. Mice were anesthetized, 1 h after the final drug administration, in order to allow blood collection from abdominal aorta. Serum was separated and stored at −20°C until assay for serum biochemical assays of BUN, Cr, and UA quantification.

### 2.8. MSU-Induced Inflammation in Rats

To investigate the anti-inflammatory activity of SM extract on gout, the experimental model was executed as mentioned previously [28]. The rats were randomly divided into 6 groups (*n* = 8): normal control group, MSU control group, colchicine group (0.3 mg/kg), and low, medium, and high dose SM extracts group (16 g/kg, 32 g/kg, and 64 g/kg raw medicinal herbs, resp.). Beside the normal control group, injected with 100 *μ*L sterile endotoxin-free saline, each group was injected with 100 *μ*L sodium urate (100 mg/mL) into the right hind paw by the intraarticular injection. Before and after 1 h of injecting sodium urate, each group was treated related sample by intragastrical administration; normal control and MSU group were treated with 0.9% normal saline. The dosage of SM extracts was selected from pilot experiments and the maximum dose was determined by its maximum solubility.

The inflammation was quantified by measuring the ankle circumference with a tape at 0 h, 2 h, 4 h, and 6 h after MSU crystal injections. At the end of the experimental period (72 h), the rats were killed by cervical decapitation. Blood from each animal was collected for plasma/serum separation 6 h after MSU crystal injections. The serum was used for testing the status of indexes such as TNF-*α*, IL-*β*, MPO, MDA, and SOD.

### 2.9. Histopathological Examination of Ankle Joints

The right hind ankle joints of rats were dissected, fixed with 10% formalin, and decalcified in EDTA for 1 month. After that, ankle joints should be dehydrated, embedded in paraffin, and then cut into 5 mm thick paraffin sections. Then, paraffin sections were stained with hematoxylin and eosin (HE) using standard techniques.

### 2.10. Biochemical Assays

The activities of TNF-*α*, IL-*β*, MDA, SOD, MPO, UA, Cr, and BUN in serum were determined photometrically in accordance with the manufacturer's protocol by using commercially available enzymatic assay kits. All the experiments described in this section were performed in triplicate to obtain means and standard deviations (SD).

### 2.11. Statistical Analysis

Data were expressed as mean ± SD. Statistical analysis was carried out using SPSS 18.0 software. Statistical significance was determined by a single-factor ANOVA test. A probability of less than or equal to 0.05 was considered to be statistically significant.

## 3. Results

### 3.1. HPLC Analysis

**Figure 1:**
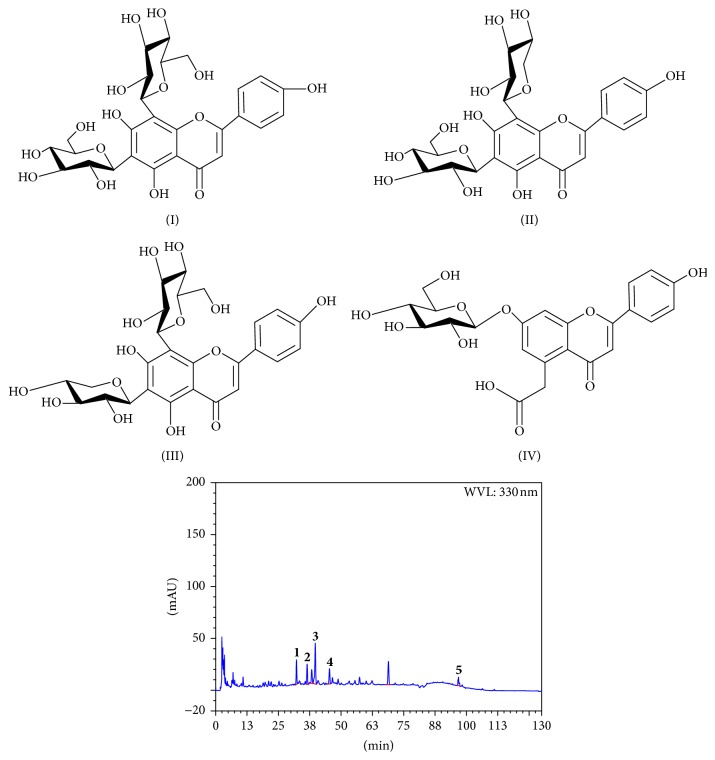
HPLC chromatogram of aqueous extract of* Selaginella moellendorffii*.** 1**: flavone-*C*-glycosides,6,8-di-*C*-*β*-D-glucopyranosylapigenin (I);** 2**: 6-*C*-*β*-D-glucopyranosyl-8-*C*-*β*-D-xylopyranosylapigenin (II);** 3**: 5-carboxymethyl-4′-hydroxyflavone-7-O-*β*-D-glucopyranoside (IV);** 4**: 6-*C*-*β*-D-xylopyranosyl-8-*C*-*β*-D-glucopyranosylapigenin (III);** 5**: amentoflavone.

HPLC chromatogram was applied for examining constituents from aqueous extract. Four flavone C-glycosides and one flavone O-glycoside have been separated and identified from SM in our research group [25, 29–31]. As reported in literature [29], the peaks of flavone-*C*-glycosides,6,8-di-*C*-*β*-D-glucopyranosylapigenin (I), 6-*C*-*β*-D-glucopyranosyl-8-*C*-*β*-D-xylopyranosylapigenin (II), 6-*C*-*β*-D-xylopyranosyl-8-*C*-*β*-D-glucopyranosylapigenin (III), 5-carboxymethyl-4′-hydroxyflavone-7-O-*β*-D-glucopyranoside (IV), and amentoflavone were marked in Figure 1. The peaks were identified by comparison with the authentic compound based on the retention time in the HPLC analysis.

**Figure 2:**
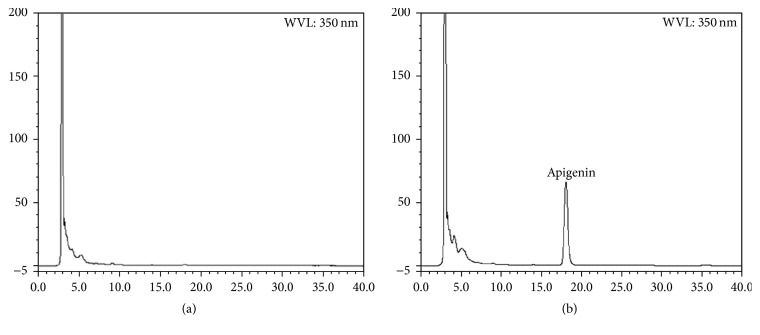
HPLC chromatogram of rat plasma after oral administration of SM extracts. (a) Blank serum. (b) The serum sample was taken at 30 min after oral administration of 30 g/kg SM extracts to rats.

The HPLC chromatogram of the plasma obtained from rats given SM extracts showed the peak which was assumed to be apigenin (Figure 2). The peak was identified as apigenin by comparison with the authentic compound based on the retention time in the HPLC analysis and U5V-VIS spectrum. The plasma apigenin concentration reached a maximum at 30 min after the administration (16 g/kg SM extracts) and began to fall from 60 min (data not shown). These results demonstrate that SM extract is absorbed and rapidly enters the circulatory system as apigenin, which is the aglycone of flavone glycosides. However, flavone glycosides were not detected in the plasma during 4 h after the administration.

### 3.2. Inhibition of XOD

The impact on XOD activity of compounds was showed in Table 1. Among these compounds apigenin was the strongest in inhibiting XOD activity whose IC_50_ value (30.0 *μ*M) is similar to positive reference substance quercetin (15.4 *μ*M), while four flavone-C-glycosides (I– IV) showed almost no inhibitory activity.

### 3.3. Antihyperuricemic Effects of SM Extract in Hyperuricemic Mice

**Figure 3:**
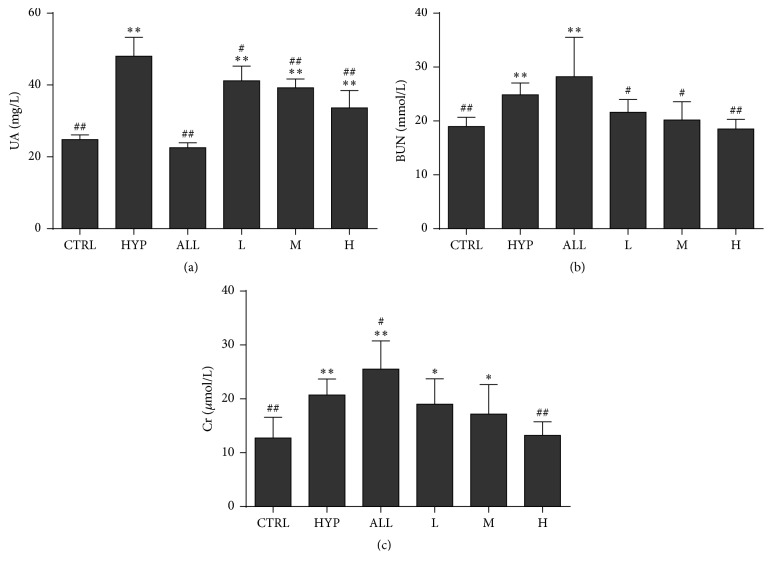
The effect of the administration of the aqueous extracts of* Selaginella moellendorffii* or allopurinol on the uric acid (a), blood urea nitrogen, (b) and creatinine (c) levels in hyperuricemic mice. Values shown are mean ± SD (*n* = 10); ^*∗*^*P* < 0.05 and ^*∗∗*^*P* < 0.01 compared with hyperuricemic control group; ^#^*P* < 0.05 and ^##^*P* < 0.01 compared to normal control group. CON: normal control group, HYP: hyperuricemic control group, ALL: allopurinol group, L: low dose* Selaginella moellendorffii* extracts group, M: medium dose group, H: high dose group, BUN: blood urea nitrogen, and Cr: creatinine.

Potassium oxonate and adenine were able to significantly increase serum urate levels compared to normal control group (Figure 3(a)). Allopurinol promoted a significant reduction on serum urate levels of hyperuricemic mice. Treatments with medium and high dose SM extract were able to significantly reduce serum urate levels compared to hyperuricemic control group (*P* < 0.01).

### 3.4. Biochemical Markers of Toxicity

The activities of BUN and Cr are sensitive indicators of kidney injury. After allopurinol treatment, plasma activity levels of BUN (Figure 3(b)) and Cr (Figure 3(c)) were increased significantly as compared with the control group (*P* < 0.01). However, levels of BUN and Cr in three dose SM extracts group were decreased as compared with hyperuricemic control group and allopurinol group, especially the high dose group which was almost the same as the control (*P* > 0.05).

### 3.5. Effect of SM Extract on Paw Edema

**Figure 4:**
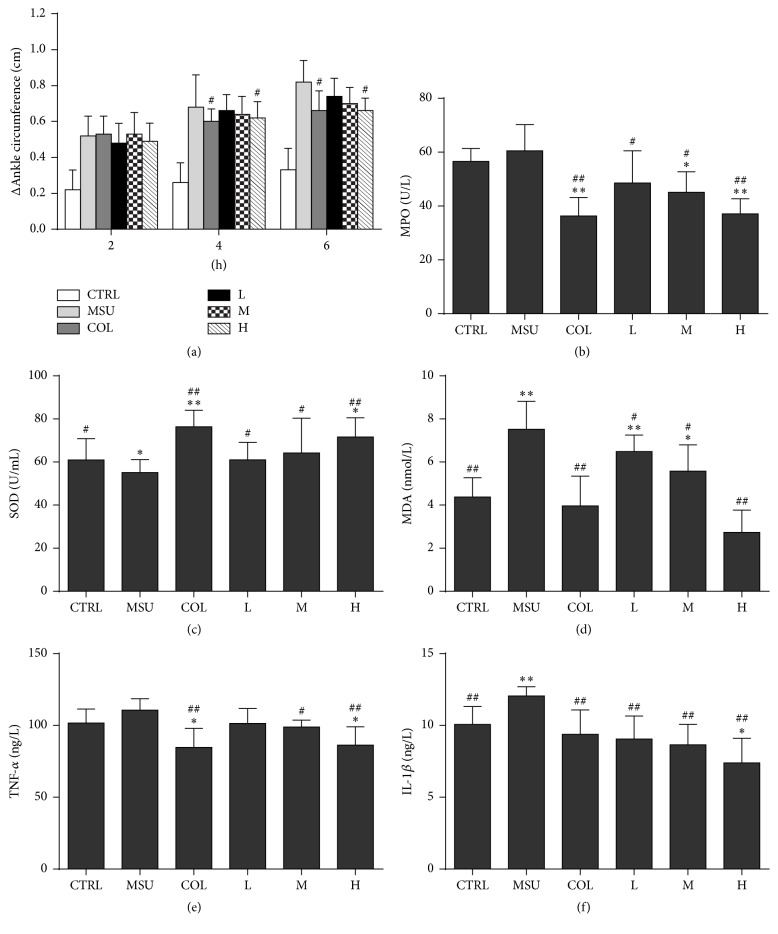
The effect of the administration of the aqueous extracts of* Selaginella moellendorffii* or colchicine on paw oedema (a), myeloperoxidase (b), malondialdehyde (c), superoxide dismutase (d), IL-1*β* (e), and TNF-*α* (f) levels in gouty arthritis rats. Values shown are mean ± SD (*n* = 10); ^*∗*^*P* < 0.05 and ^*∗∗*^*P* < 0.01 compared with MSU control group; ^#^*P* < 0.05 and ^##^*P* < 0.01 compared to normal control group. CON: normal control group, MSU: monosodium urate crystals group, COL: colchicine group, L: low dose* Selaginella moellendorffii* extracts group, M: medium dose group, H: high dose group, MPO: myeloperoxidase, MDA: malondialdehyde, and SOD: superoxide.

The paw edema of rats was roughly used to evaluate the inflammation induced by MSU crystals as a primary index.  Figure 4 portrays the effects of colchicine and SM extract on MSU crystal-induced inflammation in rats. The ankle circumference of rats in MSU group was found to be significantly increased compared to the normal group (*P* < 0.01) (Figure 4(a)). Meanwhile, the rats in SM extract group had a decrease in ankle circumference and were in dose-dependent manner. Particularly, the effect of high dose group was similar to colchicine treatment.

### 3.6. Effect of SM Extract on Serum Cytokines

MPO activity is an excellent marker of neutrophil accumulation, which is induced by the release of a variety of inflammatory mediators, such as IL-1*β* and TNF-*α*. As showed in Figure 4, the level of MPO (Figure 4(b)), TNF-*α* (Figure 4(e)), and IL-1*β* (Figure 4(f)) induced by MSU crystals had a little increase compared to that induced by normal saline, while the increase in MPO, IL-1*β*, and TNF-*α* level was prevented by both the SM extract and colchicine administration (*P* < 0.01). It was noteworthy that high dose group of SM extract showed approximately equal potential with colchicine, the most common anti-inflammatory drug used in the treatment of acute gout arthritis.

### 3.7. Effect of SM Extract on Oxidative Stress

Results showed that MSU crystal significantly increased the MDA (Figure 4(c)) but decreased the SOD (Figure 4(d)) levels in plasma when compared to control rats. SM extract and colchicine administration group showed lower MDA level but increased SOD level as compared with the MSU group (*P* < 0.01). Treatment of animals with high dose group of SM extract resulted in no significant changes in the SOD and MDA levels as compared with the colchicine group (*P* > 0.05).

### 3.8. Effect of SM Extract on Histopathological Changes in Ankle Joints

**Figure 5:**
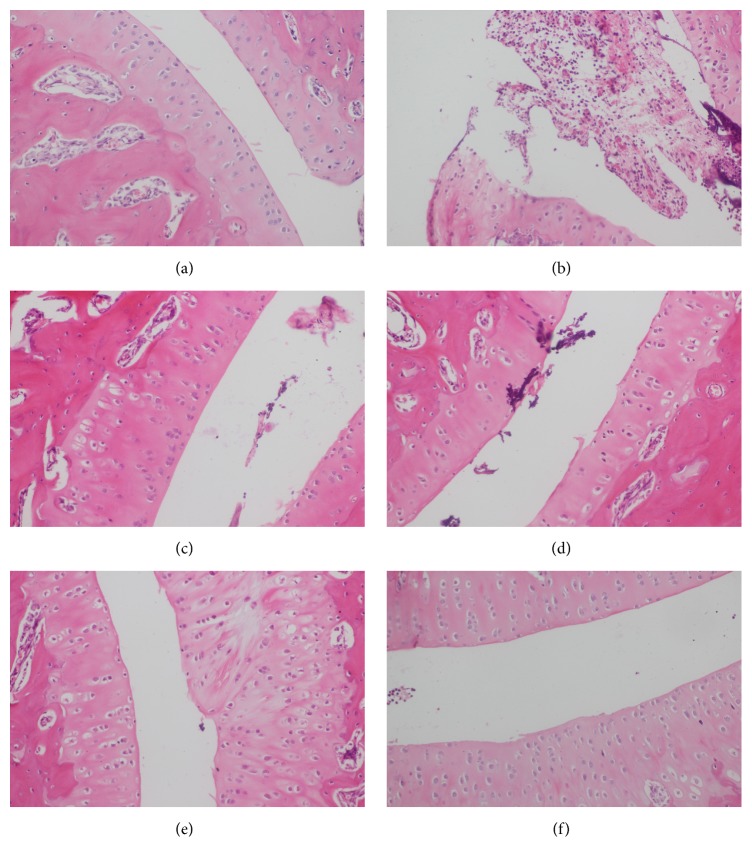
Histopathological examination of ankle joints. (a) Control normal group, (b) MSU control group, (c) colchicine group, (d) low dose* Selaginella moellendorffii* extracts group, (e) medium dose group, and (f) high dose group.

The histopathological changes in ankle joints of each group are shown in Figure 5. The cartilage and synovium in normal control group were observed in Figure 5(a); the result indicated that the intraarticular injection of sterile endotoxin-free saline could result in only a slight inflammation. In model group, a mass of neutrophil infiltration was observed (Figure 5(b)) which confirmed that MSU crystals elicited leukocyte infiltration. Yet, treatment with colchicine attenuated the neutrophil infiltration (Figure 5(c)). Not only that, our results revealed that SM extract prevented the MSU crystal-induced injury in a model of acute arthritis, which was observed in the low dose (Figure 5(d)), medium dose (Figure 5(e)), and high dose group (Figure 5(f)). In addition, the slight pathological changes in high dose group were observed.

## 4. Discussion 

### 4.1. Animal Model of Hyperuricemia and Gouty Arthritis

Gout is an inflammatory condition associated with the deposition of sodium urate crystals, leading to extreme pain in synovial joints and various tissues [32]. The treatment of inflammation and control of hyperuricemia are the major therapeutic approaches against gouty. In the present study, the experimental model of acute gouty arthritis was reproduced by injecting urate crystals and hyperuricemia was induced by potassium oxonate and adenine. Two major mechanisms have been proposed for hyperuricemia in man, excess production and insufficient metabolization of uric acid. Potassium oxonate and adenine were used to mimic both mechanisms: adenine excess production of UA and potassium oxonate impair metabolization [27]. In acute gouty arthritis model, we observed similar changes with respect to leukocyte invasion following an acute joint MSU injection. Therefore, the models are useful for studying the joint inflammation and hyperuricemia.

A slight inflammation, not significant compared with the MSU control group, existed in normal control group, because of sterile endotoxin-free saline injected. The persistence of crystals in the joints induces chronic inflammation. Unlike humans, the animals have the enzyme uricase, which catalyzes the oxidation of uric acid [33]. Hence, the acute inflammation in experimental rat model would ease up- or self-healing after about 3 days. In order to get higher drug concentration in this short experimental time, each group was treated by related drugs before and after MSU crystals injected.

### 4.2. Analysis of Antigout Constituents in SM Extract

Among the tested compounds, the strongest XOD inhibitory effect was detected for apigenin; amentoflavone exhibited only weak effects, while no inhibitory effect was found for the glucosides [26, 34]. Amentoflavone is the main constituent of SM ethyl acetate extract and aqueous extract is the traditional treatment in folk medicine [19]; thus aqueous extract is used rather than ethyl acetate extract in this research.

Four flavone C-glycosides and one flavone O-glycosides have been isolated and identified from SM n-butyl extract in our research group [24, 25, 30, 31]. After ingestion of a single dose of SM extracts, plasma peak of apigenin was short after the start of treatment, suggesting that apigenin glucosides could be deglycosylated into apigenin immediately after absorption. These data strongly indicate that the actual effect of SM aqueous extract in vivo is considerably stronger than in vitro, as the prodrug flavonoid glycosides, which were inactive in the applied enzyme assay, are transformed into the powerful xanthine oxidase inhibitor apigenin under physiological conditions [35]. As Xiao reported [36], with in vivo (oral) treatment, flavonoid glycosides showed similar or even higher antidiabetes, anti-inflammatory, antidegranulating, antistress, and antiallergic activity than their flavonoid aglycones. Flavonoid glycosides keep higher plasma levels and have a longer mean residence time than those of aglycones.

Men are at greater risk for gout than female, and 82.7% of women are postmenopausal at the time of gout onset [37]. Apigenin, a weak estrogenic flavonoid phytochemical, is found in several dietary plant foods. Extensive studies have shown that apigenin has potent antioxidant, anti-inflammatory, and anticarcinogenic and antiestrogenic properties [38]. Therefore, the main metabolites apigenin makes the medicinal plants ideal candidate for hyperuricemia, ROS, and gouty arthritis, for their activity of antioxidant, anti-inflammatory, and XOD inhibitory.

### 4.3. Mechanism of SM Extract on Antigout

Acute gouty arthritis is initially triggered by the deposition of MSU crystals into the joints space and results in the maturation of several proinflammatory cytokines and neutrophil accumulation. MSU crystal-induced inflammation is characterized by infiltration of neutrophils and release of oxygen derived free radicals, lysosomal enzymes, chemotactic factors, and proinflammatory cytokines [4]. Results of our study suggest that high dose SM extract showed significant effects in reducing hyperuricemia and MSU crystal-induced inflammation; such activity is probably due to suppress the production of proinflammatory cytokines (TNF-*α* and IL-1*β*) and the neutrophil accumulation (MPO), lower lipid peroxidation (MDA), and increase antioxidant status (SOD).

In addition, our previous study [24] had reported that SM extracts had the effect of inhibiting COX-2 and LOX expression, which could reduce the rate of AA changing into PGs and suppressing XOD activity, which is a key enzyme of UA generation.

### 4.4. Further Use of SM

Allopurinol, naproxen, or colchicine is the main drugs used to treat* gouty arthritis* and control hyperuricemia. Although these agents are generally effective, side effects are more common, such as gastrointestinal toxicity, renal toxicity, or gastrointestinal bleeding [39]. As our results showed, allopurinol had obvious kidney toxicity, but SM extract showed almost no kidney toxicity. On the other hand, it would be very interesting that one drug has both of anti-inflammatory and hypouricemic effects.

*Selaginella moellendorffii* Herba is a low perennial evergreen herb, easy to survive and breed, and could be harvested throughout the year. Although the herb powder was immersed in water and then boiled for 1 h in this study, it could be also used as tea for daily life. Our further experiment proved that prescription of traditional Chinese medicine had much more advantages (data not published). Combining with drugs with antinociceptive or suppression of the swelling might be more helpful for advanced gout patients, while SM treatment only may be better for early gout patients. These results were proved in clinic; further results will be published.

## 5. Conclusion

Apigenin glycosides, the main constituents of SM aqueous extract, might be quantitatively transformed into apigenin in vivo. Among tested compounds, the flavone aglycone apigenin exhibited by far the strongest effect on XOD in vitro. SM aqueous extract was efficient in reducing hyperuricemia and MSU crystal-induced inflammation. The curative effect of high dose was similar to the positive group, with almost no kidney toxicity. Such activity was probably due to suppressing of XOD activity, the production of proinflammatory cytokines and the neutrophil accumulation, lower lipid peroxidation, and increase of antioxidant status. Consequently, SM extracts are promising agents for the treatment of hyperuricemia and gout arthritis since they possess both antihyperuricemic and anti-inflammatory properties.

## Figures and Tables

**Table 1:** Inhibitory effects of compounds on XOD activity.

Sample number	Sample name (100 *μ*M)	Inhibition rate (%)	IC_50_ (*μ*M)
I	Flavone-C-glycosides,6,8-di-C-*β*-D-glucopyranosylapigenin	0	—
II	6-C-*β*-D-Glucopyranosyl-8-C-*β*-D-xylopyranosylapigenin	0	—
III	6-C-*β*-D-Xylopyranosyl-8-C-*β*-D-glucopyranosylapigenin	0	—
IV	5-Carboxymethyl-4′-hydroxyflavone-7-O-*β*-D-glucopyranoside	0	—
V	Amentoflavone	25.4	—
VI	Apigenin	74.8	30.0
VII	Quercetin	80.0	15.4

## Abbreviations

BUN: Blood urea nitrogen
COX: Cyclooxygenase
Cr: Creatinine
ER: Estrogen receptor
IC_50_: 50% inhibitory concentration
MDA: Malondialdehyde
MPO: Myeloperoxidase
MSU: Monosodium urate
SM: * Selaginella moellendorffii*
SOD: Superoxide dismutase
UA: Uric acid
XOD: Xanthine oxidase.
