# Corrigendum to “Pharmacological Basis for Use of* Selaginella moellendorffii* in Gouty Arthritis: Antihyperuricemic, Anti-Inflammatory, and Xanthine Oxidase Inhibition”

**DOI:** 10.1155/2018/9705495

**Published:** 2018-07-05

**Authors:** Ping Zhao, Ke-li Chen, Guo-li Zhang, Guang-rui Deng, Juan Li

**Affiliations:** ^1^Key Laboratory of Ministry of Education on Traditional Chinese Medicine Resource and Compound Prescription and College of Pharmacy, Hubei University of Chinese Medicine, Wuhan, Hubei 430065, China; ^2^Department of Pharmacy, Huanggang Hospital of Traditional Chinese Medicine, Huanggang 438000, China; ^3^State Key Laboratory of Dao-Di Herbs, National Resource Center for Chinese Materia Medica, China Academy of Chinese Medical Sciences, Beijing, China

In the article titled “Pharmacological Basis for Use of* Selaginella moellendorffii* in Gouty Arthritis: Antihyperuricemic, Anti-Inflammatory, and Xanthine Oxidase Inhibition” [1], there was a missing grant number. Accordingly, the Acknowledgments section should be corrected as follows:

This work was funded by China Postdoctoral Science Foundation (2016M601237), the Natural Science Foundation Project (30470193), the Scientific Research Foundation Project of the Education Department of Hubei Province (Q20142007), Natural Science Foundation of Hubei Province (2013CFC055), and the Young Plan Project of Hubei University of Chinese Medicine (XJ2014KJ008).
